# Stuttered swallowing: Electric stimulation of the right insula interferes with water swallowing. A case report

**DOI:** 10.1186/1471-2377-11-20

**Published:** 2011-02-05

**Authors:** Peter Sörös, Faisal Al-Otaibi, Savio WH Wong, J Kevin Shoemaker, Seyed M Mirsattari, Vladimir Hachinski, Ruth E Martin

**Affiliations:** 1 School of Communication Sciences and Disorders, University of Western Ontario, London, Ontario, Canada; 2 Department of Clinical Neurological Sciences, University of Western Ontario, London, Ontario, Canada; 3 School of Kinesiology, University of Western Ontario, London, Ontario, Canada; 4 Brain and Creativity Institute, University of Southern California, Los Angeles, California, USA; 5 Department of Physiology and Pharmacology, University of Western Ontario, London, Ontario, Canada

## Abstract

**Background:**

Various functional resonance imaging, magnetoencephalographic and lesion studies suggest the involvement of the insular cortex in the control of swallowing. However, the exact location of insular activation during swallowing and its functional significance remain unclear.

**Case presentation:**

Invasive electroencephalographic monitoring was performed in a 24-year-old man with medically intractable stereotyped nocturnal hypermotor seizures due to a ganglioglioma. During stimulation of the right inferior posterior insular cortex with depth electrodes the patient spontaneously reported a perception of a "stutter in swallowing". Stimulation of the inferior posterior insular cortex at highest intensity (4 mA) was also associated with irregular and delayed swallows. Swallowing was not impaired during stimulation of the superior posterior insular cortex, regardless of stimulation intensity.

**Conclusions:**

These results indicate that the right inferior posterior insular cortex is involved in the neural circuitry underlying the control of swallowing.

## Background

Swallowing is a complex sensorimotor function involving the coordinated activation of orofacial, pharyngeal, laryngeal, respiratory, and esophageal muscles. Although once attributed to brainstem circuits, electrophysiologic studies in animals [1], and functional brain-mapping in humans [2-4] have also implicated a distributed cortical-subcortical network in swallowing neural control involving the bilateral primary sensorimotor cortex, premotor and cingulate motor areas, frontoparietal operculum, and the insula (for review, see [5-7]). The notion of a cortical-subcortical network controlling swallowing is further supported by lesion studies in humans [8-11].

Although various lines of evidence, supported by functional resonance imaging [3,12], magnetoencephalography [13] and lesion data [8], point at the involvement of the insular cortex in the control of swallowing, the exact involvement of the insula and its functional significance remain unclear. While the right anterior insula has been particularly implicated in swallowing impairment [11,14], brain-imaging studies of swallowing have reported both anterior and posterior insular activation. A recent quantitative meta-analysis of swallow-related brain activity reported high activation likelihood in the right anterior insular cortex for water swallowing, and in the right posterior insula for voluntary saliva swallowing [15].

Studying swallowing neural control is not only important for basic neurophysiological research but also from a clinical perspective because injury to the central nervous system such as stroke frequently results in significant swallowing impairment [16]. Indeed, brain injury can give rise to severe and protracted swallowing impairment, which necessitates tube feeding. Current understanding of the neuropathophysiology of swallowing impairment, the neuroplastic mechanisms underlying swallowing recovery, as well as the principles of swallowing rehabilitation is limited (for review, see [17]).

## Case presentation

**Figure 1:**
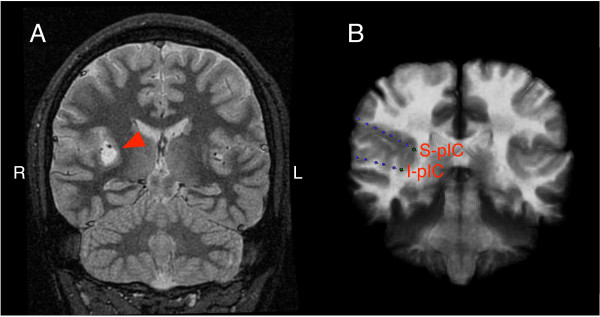
**Tumor location and electrode placement**. A) Coronal T2-weighted anatomical MRI showing a lesion with increased signal intensity in the right posterior insula marked by an arrowhead. This lesion was subsequently resected and was consistent with a ganglioglioma. B) Superimposed coronal CT and MRI images showing 3 implanted depth electrode strips in the right hemisphere. The top two lines were implanted in the insular cortex and the bottom line was inserted into the hippocampus and parahippocampus. Electrodes I-pIC (inferior posterior insular) and S-pIC (superior posterior insular cortex) were stimulated to investigate insular involvement in the cerebral control of swallowing.

Electrical stimulation of the right posterior insular cortex was performed in a 24-year-old right-handed man who developed medically intractable epilepsy at the age of 17 years and who was referred for invasive electroencephalographic monitoring and pre-surgical mapping. He was suffering from stereotyped nocturnal hypermotor seizures nearly every night. His neurological examination was normal. MRI of the brain showed a well-circumscribed tumor (18 × 12 × 11 mm) in the right posterior insular cortex (Figure 1A).

Pre-surgical electric stimulation was performed using depth electrodes that were implanted in the right posterior insular cortex, above and below the tumor (1 mm diameter size; Ad Tec Medical Instrument Corp., Racine, WI, USA) [18]. The depth electrodes were inserted stereotactically using a Leksell stereotactic frame and a Stealth station planning system (Medtronic-Sofamor Danek, Minneapolis, MN, USA). The depth electrode lines had 8 contacts (each 2 mm long) separated by 2 mm. A transopercular approach was used to implant the depth electrodes in the right insular cortex above and below the lesion. The distal electrode contacts of each depth line were located within the right posterior insular cortex. Location of the electrode contacts was confirmed by fusing a pre-implantation MRI to a post-implantation CT scan with the aid of a volumetric mutual information algorithm using the Atamai software (Atamai, Inc., London, ON, Canada; Figure 1B).

High frequency (50 Hz) bipolar electric impulses were applied at four different intensity levels (1, 2, 3 and 4 mA) with a pulse duration of 0.3 ms over 2 s (Grass Technologies, West Warwick, RI, USA). To assess the potential impact of surgery on swallowing, the patient swallowed a continuous water infusion (1 ml/s) delivered manually to the mouth via a catheter-tipped syringe. During water swallowing, electrodes S-pIC (superior posterior insular cortex) and I-pIC (inferior posterior insular cortex) were stimulated individually with increasing stimulation intensities. Swallowing was recorded from the output of a laryngeal movement sensor.

**Figure 2:**
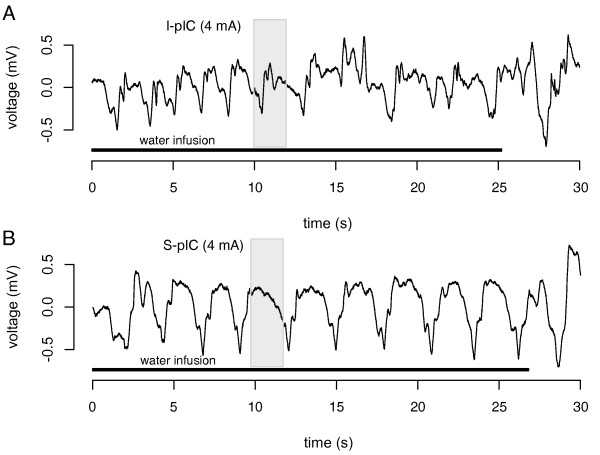
**Recordings of swallowing-related laryngeal movements**. Recording of laryngeal movements associated with water swallowing during electrical stimulation of electrode I-pIC (inferior posterior insular cortex, A) and S-pIC (superior posterior insular cortex, B), both at 4 mA. The grey area represents the electric stimulation (2 s), the black line the duration of continuous water infusion.

Stimulation with electrode I-pIC at 4 mA was associated with a dysrhythmic pattern of swallowing as indicated by irregular and delayed swallows (Figure 2A). Moreover, at all intensity levels of stimulation with electrode I-pIC, the patient spontaneously reported a perception of a "stutter in swallowing". In contrast, swallowing was not impaired during stimulation with S-pIC, regardless of stimulation intensity (Figure 2B). EEG recordings revealed that all epileptic spikes and seizures originated from the right inferior posterior insular cortex.

The tumor was surgically removed after invasive electroencephalographic monitoring and diagnosed as a ganglioglioma, a mostly benign neoplasm arising from ganglion cells. Gangliogliomas are often located in the medial and lateral temporal lobes, superior and middle temporal gyri, parahippocampal gyrus and amygdala [19], but can be found anywhere in the brain. Gangliogliomas are a frequent cause of intractable epilepsy [20]. The patient has been seizure free after removal of the ganglioglioma.

## Conclusions

The insula is a phylogenetically old, as well as cytoarchitectonically and functionally diverse area, integrating information from numerous distinct regions of the brain. Lesion studies [11,14] and functional brain imaging [12] have suggested that the anterior insular cortex is involved in the control of swallowing. The anterior insula has been implicated in the processing of visceral sensation from the pharynx [21], esophagus and gastrointestinal tract [22], vibrotactile stimulation [23], gustation [24], olfaction [25], and emotions such as disgust [26]. Other fMRI studies, however, reported swallow-related activation of the posterior insula [3], which is involved in autonomic regulation [18,27], including heart rate and respiration. Consistent with this, a meta-analysis of swallow-related brain imaging implicated both the anterior and posterior insula in swallowing [15].

Notwithstanding the possibility that slow tumor growth may have engaged compensatory swallowing mechanisms [28], the present finding that electrical stimulation of the right inferior posterior insula disrupts rhythmic water swallowing provides further evidence that the posterior insular cortex is involved in the neural circuitry underlying the control of swallowing.

Our results are consistent with previous cortical stimulation studies of the posterior insular cortex which resulted in visceral sensations and interference with swallowing [29]. Recently, detailed mapping of the insular cortex in epilepsy patients has been performed using electrical stimulation [30,31]. In 50 patients, electrical stimulation of the anterior and posterior insular cortex elicited 125 clinical responses, 22% of which involved a viscerosensory effect. Half of these viscerosensory responses consisted of sensations of unpleasant laryngo-pharyngeal constriction [30]. In one patient, spontaneous seizures originated from the right anterior insular cortex and quickly propagated to the right posterior insular cortex [30]; these seizures began with strong laryngeal discomfort and an unpleasant sensation of constriction, similar to the viscerosensory sensations elicited by electrical stimulation [30]. Another insular mapping study, again using electrical stimulation in epilepsy patients, reported that insular stimulation evoked sensations of a pharyngeal constriction and a sensation of swallowing associated with mastication [31].

The strong representation of viscerosensory afferents in the insular cortex, as indicated by electrophysiological recordings [30] and functional neuroimaging data [21], as well as the importance of upper airway sensory input for the control of swallowing [32], raises the possibility that the disruption of water swallowing observed in our patient may have been related to oropharyngeal-laryngeal sensory alterations, such as discomfort or constriction, elicited by the electrical stimulation. Although our patient did not report isolated upper airway sensations during the stimulation protocol, it is conceivable that disruption of oropharyngeal-laryngeal sensory processing may have interfered with normal water swallowing and created the impression of "stuttered swallowing". Alternatively, disruption of water swallowing in the present case may have occurred due to an effect of insular stimulation on swallowing motor execution. Further studies are required to elucidate the mechanisms underlying the swallowing phenomena documented in this clinical case.

## Competing interests

The authors declare that they have no competing interests.

## Authors' contributions

PS analyzed the data and drafted the manuscript. FAO and SWHW participated in data acquisition. JKS and SMM conceived the study, participated in its design and in data acquisition. VH participated in analysis and interpretation of the data. REM conceived and designed the study, participated in data acquisition and analysis and drafted the manuscript. All authors read and approved the final manuscript.

## Pre-publication history

The pre-publication history for this paper can be accessed here:

http://www.biomedcentral.com/1471-2377/11/20/prepub
